# Identification of disease propagation paths in two-layer networks

**DOI:** 10.1038/s41598-023-33624-y

**Published:** 2023-04-19

**Authors:** Guangjun Li, Gang Liu, Xiaoqun Wu, Lei Pan

**Affiliations:** 1 College of Sports Engineering and Information Technology, Wuhan Sports University, Wuhan, 430079 China; 2 School of Information Technology, Deakin University, Geelong, 3220 Australia; 3 School of Mathematics and Statistics, Wuhan University, Wuhan, 430072 China

**Keywords:** Information theory and computation, Statistical physics, thermodynamics and nonlinear dynamics

## Abstract

To determine the path of disease in different types of networks, a new method based on compressive sensing is proposed for identifying the disease propagation paths in two-layer networks. If a limited amount of data from network nodes is collected, according to the principle of compressive sensing, it is feasible to accurately identify the path of disease propagation in a multilayer network. Experimental results show that the method can be applied to various networks, such as scale-free networks, small-world networks, and random networks. The impact of network density on identification accuracy is explored. The method could be used to aid in the prevention of disease spread.

## Introduction

Complex network research has been a trendy area and is on the rise. The area of research is expanding, from protein interaction networks to biological networks, from scientific research networks to social networks, from information transmission networks to disease propagation networks, and from transportation networks to logistical networks. Complex network research topics include synchronization^1,2^, robustness^3^, node importance^4^, structure identification, and stability^5^, among others^6–9^. The simulation of disease models is one of them, and it is an important component of complex network research^10–12^.

Disease propagation and propagation path identification are two aspects of research on the disease model of a complex network. The single-layer network model of disease was first investigated in disease models^13^. The models involved include SIS (susceptible-infected-susceptible)^10–13^, SIR (susceptible-infected-removed)^12,14^, SIRS (susceptible-infected- removed- susceptible)^15^, SEIR (susceptible-exposed-infected-recovered)^16^, SEIRS (susceptible-exposed-infected-recovered-susceptible)^17^, SIQRS (susceptible-infected-quarantined-recovered-susceptible)^18^, SIVRS (susceptible-infected-variant-recovered-susceptible)^19,20^, etc. The propagation characteristics of the SIS model under the condition of infected individual mobility^10^, the nonlinear infectivity and adaptive weighted SIS model^13^, the network-based SIS epidemic model with global behavior notes^21^, the SIS epidemic model with infectious latency^22^, the epidemic situation of nodes with a square lattice^13^, and so on are among the most studied models. Path identification in the SIS model of a single-layer disease propagation network was also investigated by Zhesi Shen et al.^23^. Dongmei Fan et al. investigated the influence of geometric correlation on epidemic propagation with multilayer disease models^24^. Paulo Cesar Ventura da Silva et al. investigated epidemic propagation with consciousness and varied time scales in multilayer networks^25^, and Fuzhong Nian et al. simulated the MR-SIS propagation process in multirelational networks^26^. There is more research on the model of disease propagation in the above research than there is on identifying the propagation path. There is currently just a small amount of literature on disease path identification, and it is focused on single-layer disease networks. In multilayer networks, there is a lack of literature on path identification. This may be because path identification in multilayer disease networks is difficult. Furthermore, identifying the disease propagation path in multilayer networks is critical for disease prevention and control^27^. We will investigate the identification of propagation paths in a multilayer disease network in this paper.

The Granger causality test^28^, synchronization-based^29^, and compressive sensing-based topology identification^30,31^ are all common methods. The Granger method of topology identification necessitates stochastic network node perturbations, which is not realistic in disease propagation networks. The synchronization-based method requires constructing an auxiliary network with a very general form and designing some adaptive controllers. However, the compressive sensing-based method in this paper does not need these additional conditions. To identify the topology of a disease propagation network, only a small amount of measurement data is needed. Compressed sensing is a new sampling theory that can obtain discrete samples of a signal when the sampling rate is substantially lower than Nyquist, allowing for distortion-free signal reconstruction.The compressive sensing-based method has the advantages of requiring less data to be measured and having a high identification accuracy^32,33^. The basic concept of compressive sensing is $Y=AX$, where $Y\in R^m$, *A* is a $m\times n$ dimensional matrix, $X\in R^n$, and $m\ll n$. Only a few values of *Y* and *A* need to be measured to reliably identify *X* when there are enough zero elements in it. The aim of this paper is to use compressive sensing to identify the topology of disease spread in multilayer networks.

**Figure 1:**
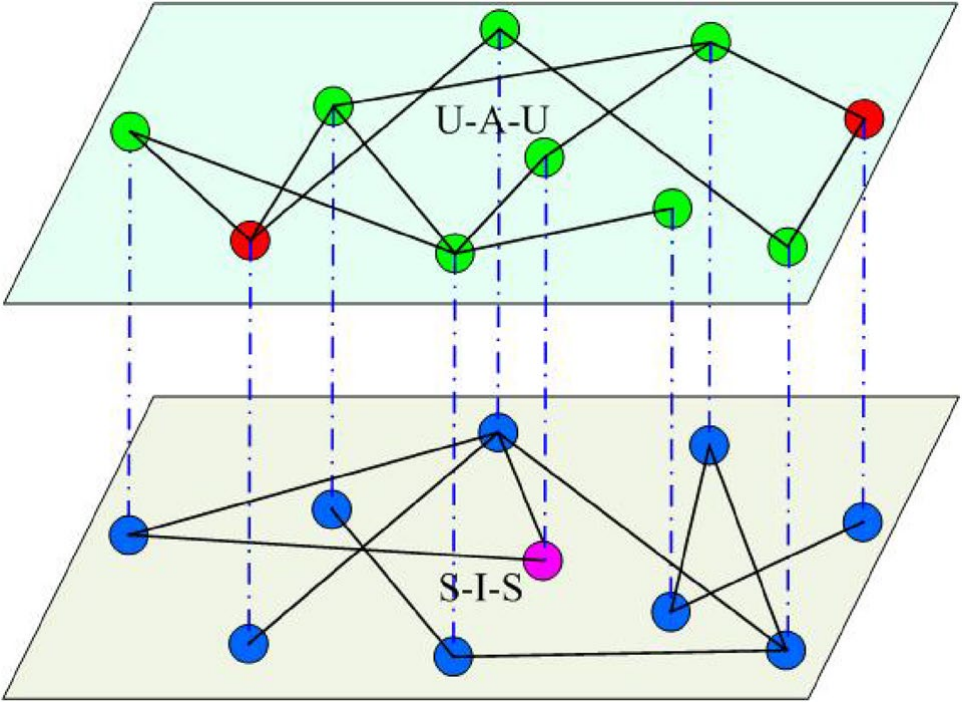
Sketch of a two-layer epidemic network used in this work. The upper layer (virtual contact) represents the spreading of awareness, where nodes have two possible states: unaware (U) or aware (A). The lower layer (physical contact) corresponds to the network where the epidemic spreading takes place. The nodes are the same actors as that in the upper layer, and their states can be: susceptible (S) or infected (I).

## Method

Figure 1 depicts a typical two-layer disease model. The crowd’s spread of consciousness is shown in the upper layer^34^, where nodes represent the individuals and edges between nodes indicate information propagation paths. Contacts via Facebook, WeChat, and other social media platforms can be used to spread information. The model used in the upper layer is UAU, where U represents the individual who is unaware of the disease and A represents the one who is aware. After acquiring information from those who are aware, those who are unaware can be changed into aware, with a probability of $\lambda$. The state of the node is A (aware) if the node is aware of disease; otherwise, the status is U (unaware). The lower layer depicts the spread of disease among humans, with nodes representing people and edges between nodes representing disease propagation paths. We use the conventional SIS model in the lower layer, where S represents the susceptible population and I represents the infected population. In the lower layer, we use *S*(*i*) to denote the status of infected and susceptible nodes as follows:

1 $$\begin{aligned} S(i)=\left\{ \begin{array}{ll} 1~~~~~ &{}\hbox {infected},\\ 0~~~~~~&{} \hbox {susceptible}, \end{array}\right. \end{aligned}$$

and the state of an arbitrary node *i* is denoted as *M*(*i*) in the upper layer, where

2 $$\begin{aligned} M(i)=\left\{ \begin{array}{ll} 1~~~~~ &{}\hbox {aware},\\ 0~~~~~~&{} \hbox {unaware}. \end{array}\right. \end{aligned}$$

We use $a_{ij}$ and $b_{ij}$ in the networks to denote the edges between nodes in the upper and lower networks, respectively (especially if there is an edge between node *i* and node *j*, then $a_{ij} = 1$ or $b_{ij} = 1$; otherwise, $a_{ij} = 0$ or $b_{ij} = 0$). If node *i* is unaware of the disease during disease propagation, node *i* may be converted to aware after being notified by surrounding nodes. The probability is denoted by $r_i(t)$. The probability that node *i* will be infected by surrounding nodes at time *t* is $q_i^{A01}(t)$ if it is susceptible and aware. The probability is $q_i^{U01}(t)$ if node *i* is unaware. The following are the changes in $r_i(t)$, $q_i^{A01}(t)$, and $q_i^{U01}(t)$ over time:

3 $$\begin{aligned} r_i(t)= & {} 1-(1-\lambda )^{{a_{i1}{M_{1}(t)}}+\cdots +{a_{iN}{M_{N}(t)}}}=1-(1-\lambda )^{\sum \limits _{j=1,j\ne i}^N {a_{ij}M_{j}(t)}},\nonumber \\ q_i^{A01}(t)= & {} 1-(1-p_i^A)^{{b_{i1}{S_{1}(t)}}+\cdots +{b_{iN}{S_{N}(t)}}}=1-(1-p_i^A)^{\sum \limits _{j=1,j\ne i}^N {b_{ij}S_{j}(t)}},\nonumber \\ q_i^{U01}(t)= & {} 1-(1-p_i^U)^{{b_{i1}{S_{1}(t)}}+\cdots +{b_{iN}{S_{N}(t)}}}=1-(1-p_i^U)^{\sum \limits _{j=1,j\ne i}^N {b_{ij}S_{j}(t)}}, \end{aligned}$$

where $p_i^A$ is the infection probability of node *i* in the susceptible population if node *i* is aware, $p_i^U$ is the infection probability of node *i* if node *i* is unaware, and $\lambda$ is the probability of node *i* transitioning from unaware to aware when getting a notification. Meanwhile, node *i* recovers at a probability of $\sigma$ after infection.

Taking the logarithm of both sides of Eq. (3), we can obtain

4 $$\begin{aligned} \begin{array}{ll} &{} \hbox {In}(1-q_i^{A01}(t))=\hbox {In}(1-p_i^A)\cdot \sum \limits _{j=1,j\ne i}^N{b_{ij}S_{j}(t)}, \\ &{}\hbox {In}(1-q_i^{U01}(t))=\hbox {In}(1-p_i^U)\cdot \sum \limits _{j=1,j\ne i}^N{b_{ij}S_{j}(t)}. \end{array} \end{aligned}$$

When one measures at different time points and obtains the state of multiple copies, then Eq. (4) can be written as a matrix:

5 $$\begin{aligned} \Phi _{m\times (N-1)}X_{(N-1)\times 1}=Y_{m\times 1}, \end{aligned}$$

where $\Phi _{m\times (N-1)}$ is defined as the state of other nodes except node *i*.

$$\begin{aligned} X= & {} (b_{i1}, b_{i2}, \cdots , b_{i-1}, b_{i+1}, \cdots , b_{N})^T;\\ Y= & {} (y_1,y_2, \cdots , y_m)^T, y_i=\hbox {In}(1-q_i^{A01}(t))~~~{\hbox {or}}~~~y_i=\hbox {In}(1-q_i^{U01}(t));\\ \Phi= & {} (\phi _1, \phi _2, \cdots , \phi _m)^T;\\ \phi _i= & {} (S_1(t_i)\hbox {In}(1-p_i^{t_i}),\cdots , S_{i-1}(t_i)\hbox {In}(1-p_i^{t_i}), S_{i+1}(t_i)\hbox {In}(1-p_i^{t_i}), \cdots ,\\{} & {} S_{N}(t_i)\hbox {In}(1-p_i^{t_i} )) \end{aligned}$$

at different time *t*, one obtains the following equation:

6 $$\begin{aligned} \begin{array}{ll} &{} \left( \begin{array}{cccccc} S_1(t_1)\hbox {In}(1-p_i^{t_1})&{} \cdots &{} S_{i-1}(t_1)\hbox {In}(1-p_i^{t_1}) &{} S_{i+1}(t_1)\hbox {In}(1-p_i^{t_1}) &{} \cdots &{} S_N(t_1)\hbox {In}(1-p_i^{t_1})\\ S_1(t_2)\hbox {In}(1-p_i^{t_2})&{} \cdots &{} S_{i-1}(t_2)\hbox {In}(1-p_i^{t_2}) &{} S_{i+1}(t_2)\hbox {In}(1-p_i^{t_2}) &{} \cdots &{} S_N(t_2)\hbox {In}(1-p_i^{t_2})\\ \vdots &{} \vdots &{}\vdots &{}\vdots &{} \vdots &{} \vdots \\ S_1(t_m)\hbox {In}(1-p_i^{t_m})&{} \cdots &{} S_{i-1}(t_m)\hbox {In}(1-p_i^{t_m}) &{} S_{i+1}(t_m)\hbox {In}(1-p_i^{t_m}) &{} \cdots &{} S_N(t_m)\hbox {In}(1-p_i^{t_m})\\ \end{array}\right) \\ &{} \times \left( \begin{array}{c} b_{i1}\\ \vdots \\ b_{i,j-1}\\ b_{i, j+1}\\ \vdots \\ b_{iN} \\ \end{array}\right) = \left( \begin{array}{c} \hbox {In}(1-q_i^{t_1{01}}(t_1))\\ \hbox {In}(1-q_i^{t_2{01}}(t_2))\\ \vdots \\ \hbox {In}(1-q_i^{t_m{01}}(t_m)) \end{array}\right) . \end{array} \end{aligned}$$

We just need to solve vector *X* in Eq.(5) to find the propagation paths between node *i* and other nodes in the disease model. Letting $i= 1, 2,\cdots , N$, we can identify the paths among all nodes. The problem is then transformed into solving the equation $\Phi X=Y$, where $\Phi$ is a $m\times (N-1)$ matrix, *Y* is a known vector, and the *X* vector is to be determined. According to the literature^23^, $q_i^{A01}(t)$ and $q_i^{U01}(t)$ can be obtained based on big data. For a specific disease, $p_i^A$ and $p_i^U$ are known^23^, and only the status of each node needs to be measured. When there are few nonzero elements in *X*, we only need a minimal number of measurements to accurately solve *X*, according to compressive sensing theory^35,36^. Minimizing the number of nonzero components in *X* produces the sparsest solution to $\Phi X=Y$ with respect to *X*, i.e.,

7 $$\begin{aligned} \min \parallel X\parallel _0, \hbox {subject~~ to ~~}\Phi X=Y. \end{aligned}$$

Classical Tikhonov regularization is used to solve $\Phi X=Y$ to obtain accurate reconstruction and increase numerical stability. Then, this problem can be approximated by

8 $$\begin{aligned} \min \limits _X \parallel \Phi X-Y\parallel _2+\alpha \parallel X\parallel _1, \end{aligned}$$

The regularization value $\alpha$ is used to avoid large deviations from the optimal solution. To solve the convex optimization problem, we usually use alternating direction method of multipliers (ADMM) algorithms^37^.

### Numerical simulation

We use different network structures for simulation to verify the universality of our method of identifying propagation paths. It is assumed that a specific disease has spread throughout a community. The notations $p_i^A$ and $p_i^U$ denote the infection probability of a node in the aware and unaware states, respectively. In practice, $p_i^A<p_i^U$. $\lambda$ represents the probability that a node will become aware of the disease after being notified by any of its neighbours, and $\sigma$ represents the recovery probability. We set the path value to 1 if there is a disease propagation path between two nodes in the community, which corresponds to $b_{ij}=1$. We state in the identification procedure that the path is regarded to exist if the identification value is $b_{ij}\in [1-\varepsilon , 1+\varepsilon ]$ and nonexistent if the value is $b_{ij}\in [-\varepsilon , \varepsilon ]$. The value of $\varepsilon$ in this paper is 0.01.

TPR (true positive rate) and TNR (true negative rate) are indicators of identification accuracy, with the TPR being the ratio of all correctly identified paths out of all existing paths and the TNR representing the percentage of all correctly identified nonexistent paths out of all nonexistent paths.

The identification error for nonzero (existing) and zero (nonexistent) edges is represented by $E_{nz}$ and $E_{z}$, respectively.

$$\begin{aligned} E_{nz}=\sum \limits _{i=1,j=1}^N\sum _{j\ne i, b_{ij}=1}^N{({b_{ij}-b_{ij}^{'}})}/{\sum \limits _{i=1,j=1}^N\sum _{j\ne i, b_{ij}=1}^N{b_{ij}}}, \end{aligned}$$

where $b_{ij}^{'}$ indicates the identification value of the existing edge and $b_{ij}$ indicates the true value. The true value of $b_{ij}$ for the existing edge between nodes *i* and *j* is 1.

$$\begin{aligned} E_{z}=\sum \limits _{i=1,j=1}^N\sum _{j\ne i, b_{ij}=0}^N b_{ij}^{'}/{\sum \limits _{i=1,j=1}^N\sum _{j\ne i, b_{ij}=0}^N 1}, \end{aligned}$$

where $b_{ij}^{'}$ indicates the identification value of nonexistent edges.

#### Identification paths of disease propagation in ER, WS, and BA networks

**Figure 2:**
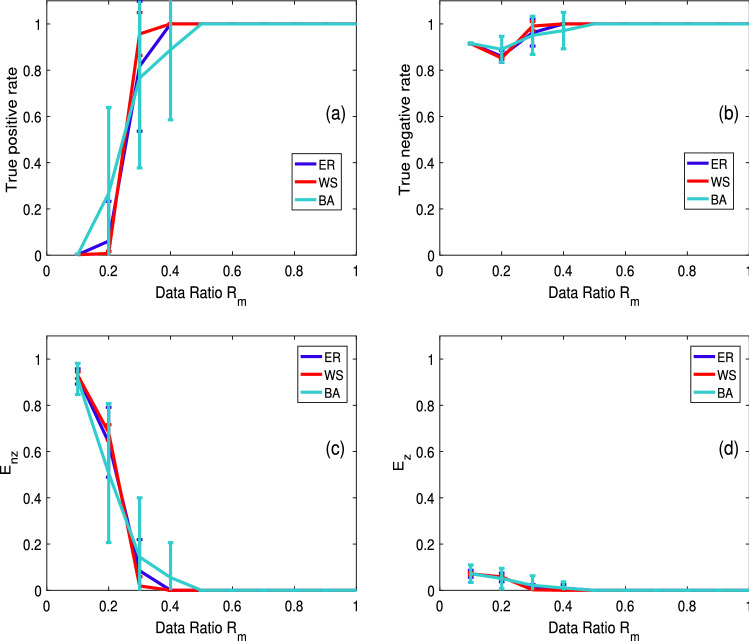
Identification results of disease propagation paths in three different networks: ER, WS, and BA. (**a**) True positive rate (**b**) True negative rate. (**c**) Average relative error (**d**) Average absolute error.

We initially discover disease propagation paths in various types of networks using numerical simulation. Random networks (ER), small-world networks (WS), and scale-free networks (BA) are the networks we chose. The three networks are mostly used to mimic real population relationships in disease propagation models^6,27,34^. The following are the practical implications: Everyone can be considered a node, and there are a great number of paths linking them in a WS network. People who know each other are represented by the connected nodes. A few nodes in BA networks have a great number of connections, while the majority of nodes have minimal connections.Given a specific number of nodes, there is the same probability of a path existing between each pair of nodes in an ER network, *u* and *v*. The data ratio in the simulation refers to the proportion of actual observed data to the data necessary for a typical solution. The typical solution takes $N-1$ measurements to solve the solution vector $(b_{i1}, b_{i2}, \cdots , b_{i-1}, b_{i+1}, \cdots , b_{N})^T$, as given in Eq. 6. However, compressive sensing assumes that only *k* times ($k<N-1$) of data measurement are necessary to solve the vector, and the data ratio is defined as the ratio of *k* to $N-1$. The parameters are as follows: all networks’ edge connection probability is $10\%$, and the total number of nodes in all three networks is 500. The infection probability is $20\%$, $p_i^A=0.4, p_i^U=0.7$, and $\sigma$=0.2. The identification of paths connecting all nodes is repeated separately 30 times in the simulation, and the average value is taken. The result of the identification is presented in Fig. 2, with the top and lower boundaries of each data point label representing the result’s standard deviations.

The disease propagation paths for the three networks can be accurately determined, as shown in Fig. 2. The WS network outperforms the others in terms of identification, while the ER network comes in second. This could be because the nodes in the WS network are more uniformly connected than those in other networks. When the data ratio is $40\%$, the propagation paths of the WS and ER networks may be reliably identified in Panels (a) and (b) of Fig. 2. The paths of the BA network are also accurately identified when the data ratio is $50\%$. The errors of identification vary with the data ratio, as seen in Panels (c) and (d) of Fig. 2. The relative error of the real existing path is shown in Panel (c) of Fig. 2, whereas the average absolute error of the nonexistent path is shown in Panel (d). When the data ratio is $40\%$, the average relative error and absolute error of the WS and ER networks in identification are already very small, as shown in Panels (c) and (d) of Fig. 2. The ER network is zero and 0.009, and the WS network is all zero. The BA network is the worst. When the data ratio is $50\%$, each of the three networks has a zero error.

#### Impact of network density on identification

**Figure 3:**
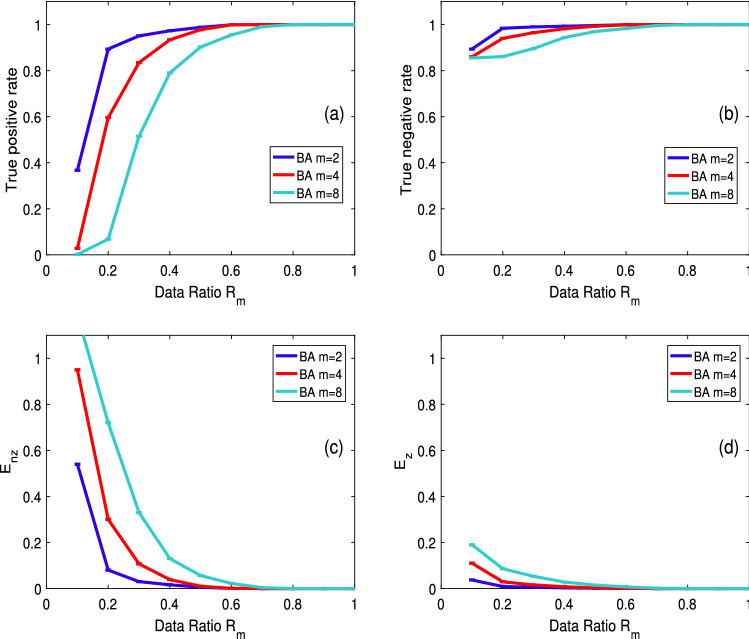
Identification performance with varying data ratio $R_m$ in three 100-node BA networks, where $\varepsilon =0.01$, and the density parameter is given as $m=2$, 4, and 8 separately.

To investigate the impact of network density on identification, we simulate networks of the same kind but with varied densities. In this case, we use the BA scale-free model. The network’s density parameters are $m = 2$, $m = 4$, and $m = 8$. When generating the network, *m* represents the number of connection edges of the new node. The network density is $3.92\%$, $7.68\%$ and $14.72\%$, respectively. The number of network nodes is set to 100, and the rest of the settings are the same as in “Conclusion” Section Fig. 3 depicts the identification result.

As shown in Fig. 3, propagation paths may be accurately identified at three different path densities while maintaining the same data ratio. However, as the path density of the network increases, the accuracy of the identification decreases. The identification accuracy of the TPR in three different path densities is $90\%$, $60\%$, and $7\%$ when the data ratio is $20\%$, as shown in Panel (a) of Fig. 3. The identification accuracy of the TNR is better than that of the TPR, as shown in Panel (b) of Fig. 3; however, the identification accuracy diminishes as path density increases. The identification accuracy is $98\%$, $94\%$, and $86\%$ when the data ratio is $20\%$. The relative and absolute errors of identification rapidly decrease as the data ratio increases, as seen in Panels (c) and (d) of Fig. 3. The absolute error is decreased to zero when the data ratio is $60\%$, and the relative error is reduced to zero when the data ratio is $70\%$.

The compressive sensing method has the advantage of identifying the unknown path with less data. Only a few monitoring data are required to determine the disease’s propagation path. Even with a poor data ratio, we can still roughly identify the path that the disease takes to propagate. Fig. 3 illustrates this. We only need $20\%$ of the data in the BA network with $3.92\%$ density, and the recognition accuracy can reach $90\%$. When the network density is $14.72\%$, the required monitoring data are approximately $40\%$, and the identification accuracy is approximately $80\%$.

## Conclusion

To accurately identify disease propagation paths in multilayer networks, this paper uses compressive sensing to identify disease propagation paths using only a few measurement data. The method can accurately identify the path in many types of networks, including ER, WS, and BA, according to experimental data. It has the best identifying performance among them in the WS network. The identification performance of BA networks with various densities has been examined and assessed. The results reveal that as network density increases, the accuracy of identification decreases and the error increases. The path of disease propagation can still be accurately identified assuming appropriate measurement data are added. This method could help in the prevention of disease epidemics in the general population.

## Data Availability

The datasets used and analysed during the current study available from the corresponding author on reasonable request.
